# Advances in the Anti-Tumor Activity of Biflavonoids in *Selaginella*

**DOI:** 10.3390/ijms24097731

**Published:** 2023-04-23

**Authors:** Mengdie Ren, Sihui Li, Qiong Gao, Lei Qiao, Qianping Cao, Ze Yang, Chaoqiang Chen, Yongmei Jiang, Gang Wang, Shaobin Fu

**Affiliations:** 1 School of Pharmacy, Zunyi Medical University, Zunyi 563000, China; 13118587930@163.com (M.R.); lisihui0318@163.com (S.L.); 18908569330@163.com (L.Q.); caoqianping1993@163.com (Q.C.); yangze7380@163.com (Z.Y.); 18705598336@163.com (C.C.); 18385042506@163.com (Y.J.); 2 Key Laboratory of Basic Pharmacology of Ministry of Education and Joint International Research Laboratory of Ethnomedicine of Ministry of Education, School of Pharmacy, Zunyi Medical University, Zunyi 563003, China

**Keywords:** *Selaginella*, biflavonoids, anticancer, in vitro and in vivo

## Abstract

Despite the many strategies employed to slow the spread of cancer, the development of new anti-tumor drugs and the minimization of side effects have been major research hotspots in the anti-tumor field. Natural drugs are a huge treasure trove of drug development, and they have been widely used in the clinic as anti-tumor drugs. *Selaginella* species in the family *Selaginellaceae* are widely distributed worldwide, and they have been well-documented in clinical practice for the prevention and treatment of cancer. Biflavonoids are the main active ingredients in *Selaginella*, and they have good biological and anti-tumor activities, which warrant extensive research. The promise of biflavonoids from *Selaginella* (SFB) in the field of cancer therapy is being realized thanks to new research that offers insights into the multi-targeting therapeutic mechanisms and key signaling pathways. The pharmacological effects of SFB against various cancers in vitro and in vivo are reviewed in this review. In addition, the types and characteristics of biflavonoid structures are described in detail; we also provide a brief summary of the efforts to develop drug delivery systems or combinations to enhance the bioavailability of SFB monomers. In conclusion, SFB species have great potential to be developed as adjuvant or even primary therapeutic agents for cancer, with promising applications.

## 1. Introduction

New growths produced by the expansion of local tissue cells in response to many tumorigenic triggers are referred to as “tumors”. It is primarily a mass protrusion that occupies space, also known as a neoplasm [1,2]. In terms of morbidity and mortality, cancer continues to be a major global public health issue, coming in second place only behind cardiovascular disease [3,4]. A report on worldwide cancer statistics published in 2020 by the International Agency for Research on Cancer indicated that, in this century, cancer might surpass cardiovascular disease as the major cause of premature death in the majority of countries. By 2040, there are projected to be 28.4 million new cases of cancer worldwide, an increase of 47% from 2020 [5,6]. The pathogenic mechanisms of cancer include maintaining proliferative signals, eluding growth inhibitors, avoiding cell death, establishing replicative immortality, initiating angiogenesis, and triggering invasion and metastasis [7]. Over the last 50 years, a lot of strategies have been employed to slow the spread of cancer, namely surgery, radiotherapy, and systemic therapy [8,9]. These treatments, however, have numerous limitations and side effects, such as a high incidence of drug resistance and multidrug resistance, low efficacy of some targeted therapies, and severe adverse responses, whether administered alone or in combination [10,11]. Thus, developing novel anti-tumor medications and minimizing side effects have consistently been major areas of research in the anti-tumor field.

Due to their multi-channel and multi-target characteristics, anti-tumor compounds derived from natural products are typically used to treat advanced cancer and relieve early cancer symptoms. Herbs used in traditional medicine are the source of most natural products [12,13,14]. Over the past few decades, natural products have been a significant source for the development of new anti-tumor drugs [11]. Currently, more than 100 natural compounds are clinically used to treat cancers [15,16]. For example, studies have demonstrated that the active ingredient in *Curcuma longa*, curcumin, exerts anti-tumor effects by boosting apoptosis, inhibiting cell proliferation, obstructing tumor angiogenesis and metastasis, and inducing autophagy [17,18]. For example, paclitaxel exerts its anti-breast cancer effects by blocking mitosis (affecting B-cell lymphoma 2 (Bcl-2) phosphorylation), controlling microtubule polymerization, affecting calcium signaling, and regulating microRNA expression profiles [19]. Furthermore, significant anti-cancer efficacy is exhibited by artemisinin, which can generate reactive oxygen species in cancer cells, induce cell cycle arrest and autophagy, block cancer cell invasion and migration, and accelerate cancer cell apoptosis [20].

In recent years, a lot of literature has been reported about the classical anti-tumor signaling pathways of natural products, which are found to have the advantages of multiple targets and pathways, providing a sufficient theoretical basis for natural products as more promising antitumor drugs. For instance, the phosphoinositide 3-kinase (PI3K) signaling pathway is a crucial signaling pathway for normal cell physiological metabolism. By inhibiting this signaling pathway, tumor growth and metastasis can be prevented. Activation of PI3K can also assist in the phosphorylation of Akt and, ultimately, together play a crucial role in tumor progression [21]. For instance, Yan et al. [22] found that baicalein induced apoptosis and autophagy in breast cancer cells by inhibiting PI3K/Akt signaling pathway. In addition to the PI3K signaling pathway, the signal transducer and activator of the transcription 3 (STAT3) signaling pathway is one of the key targets for cancer therapy, which is a significant intracellular signal transduction protein and transcription factor directly related to the growth of tumors [23]. Luo et al. [24] found that Bavachin induces iron death in osteosarcoma cells via inhibition of STAT3 activity. Additionally, mitogen-activated protein kinase (MAPK) is a crucial intracellular signal transduction system that controls a variety of processes in cells, including cell growth, proliferation, differentiation, apoptosis, adhesion, and migration. These processes have an impact on drug resistance, invasion, and metastasis, as well as tumorigenesis, making it one of the potential targets of anti-tumor medications [25].

**Figure 1:**
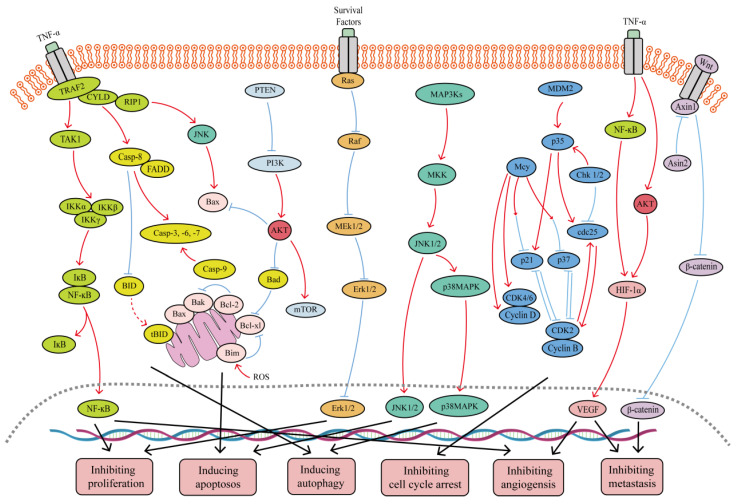
Major signaling pathways involved in the anticancer effects of SFB. SFB can inhibit the TNF-α/NF-κB, PI3K/Akt, Wnt/β-catenin, Ras/MEK/Erk, and PI3K/Akt/mTOR signaling pathways. In addition, SFB can activate the JNK1/2, p38 MAPK and mitochondrion-mediated caspase-dependent apoptotic signaling pathways. Red curves indicate inhibition, and blue arrows indicate activation of these processes.

With more than 700 species recognized, the genus *Selaginella* of the family *Selaginellaceae* is extensively distributed around the world; especially, it is primarily found in tropical and subtropical areas. These species have been reported to possess many bioactivities that include anticancer, anti-inflammatory, antibacterial, antiviral, antioxidant, anti-aging, hypoglycemic, and other activities [26,27]. However, the current pharmacological studies of *Selaginella* plants are mainly focused on anti-tumor effects. For example, Ethyl acetate fractions from *Selaginella doederleinii* exhibited cytotoxicity effects for A549 cell lines, 7721 cell lines, Hela cell lines and Eca-109 cell lines, among which it has the strongest inhibitory effect on Hela cell lines with IC50 value of 37.53 μg/mL [28]. Li et al. [29] discovered that ethyl acetate extract from *Selaginella doederleinii* induced autophagic death and apoptosis in colorectal cancer cells through the PI3K-Akt-mTOR and AMPKα signaling pathways. Lei J et al. [30] found that the biflavonoid extracts from *S. moellendorffii* exhibited a noticeable negative effect on the growth rate of HCT-116 cell lines and HeLa cell lines in the range of 0 μg/mL to 500 μg/mL. Qin et al. [31] used proline-lactic acid to prepare biflavonoid extracts, which showed significant inhibitory activity against tumor A549 cells, sw1990 cells and HepG2 cells. Therefore, we comprehensively discussed biflavonoids from *Selaginella*, which are the main active ingredient that exerts anti-tumor effects and devoted ourselves to elucidating its anti-tumor mechanism of action and its signaling pathway in the review. As shown in Figure 1.

In the last two decades, scientists have discovered that *Selaginella* plants have a wide range of active components, mostly classified as alkaloids, selaginellins and phenolic acids. Selaginellin components have been found to have potential anti-tumor activity due to their unique triple-bond structure. For example, Zhang et al. [32] isolated five selaginellin derivatives from *Selaginella tamariscina*, and two of the new selaginellins were structurally characterized by spectroscopic analysis. Cytotoxic activity assessment showed that the two new selaginellins exhibited moderate toxicity against human cancer cell lines (U251, HeLa, MCF-7). In addition, Thamnarak et al. [33] isolated two new ortholignans siamensinols (**1**–**2**) and seven known compounds agatharesinol (**3**), syringaresinol-glucoside (**4**), noreugenin from *Selaginella siamensis* (**5**), 8-methyleugenitol (**6**), melachromone (**7**), uncinoside A (**8**), and daucosterol (**9**). Among them, compounds **1**–**2** showed moderate inhibitory effects on MOLT-3 cells, while compounds **6**–**8** showed moderate inhibitory effects on three tumor cells (HepG2, A549 and HuCCA-1).

## 2. Structural Characteristics of *Selaginella* Biflavonoid

**Figure 2:**
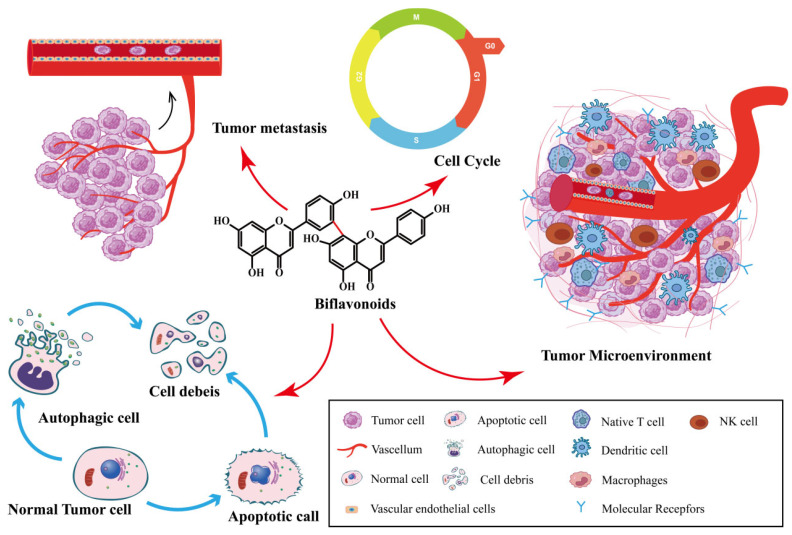
Synopsis of the mechanisms of SFB action against tumor cells.

Biflavonoids are the primary active ingredients of *Selaginella* plants, exerting anti-cancer effects [26]. SFB species exert anti-tumor effects by inhibiting cancer cell proliferation, inducing cancer cell apoptosis, inhibiting tumor metastasis and angiogenesis, inducing autophagy, and impacting the tumor microenvironment. As shown in Figure 2. SFB has been shown to have anti-cancer effects on a variety of tumors, including ovarian cancer, lung cancer, prostate cancer, breast cancer and digestive system tumors.

**Figure 3:**
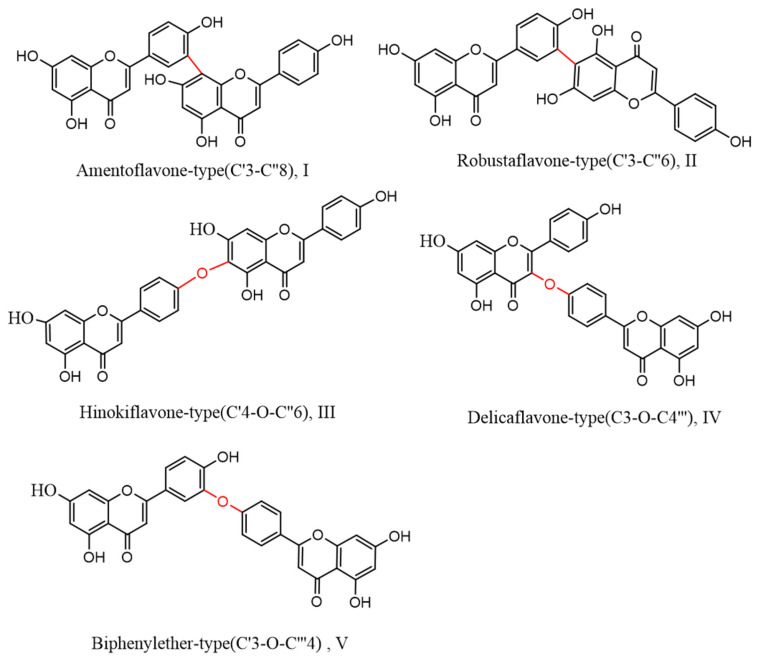
Biflavonoid structure types from the genus *Selaginella*.

Biflavonoids, a unique class of flavonoids, are primarily soluble in ethyl acetate. Their basic structural unit is made up of two molecules of flavonoids, generally in accordance with a C–C or C–O–C link. Currently, 71 different types of biflavonoids have been discovered in *Selaginella* plants [34]. They can be divided into five categories based on the various connection methods. As shown in Figure 3: ① amentoflavone-type (C′3-C″8)(I), of which there are 24 species, mainly represented by amentoflavone and isoginkgetin; ② robustaflavone-type (C′3-C″6)(II), with 23 species, mainly represented by robustaflavone; ③ hinokiflavone-t (C′4-O-C″6)(III), with ten species, mainly represented by hinokiflavone and isocryptomerin; ④ delicaflavone-type (C3-O-C4‴)(IV), of which there are four species, mainly represented by delicaflavone; and ⑤ biphenylether-type (C′3-O-C‴4)(V), of which there are two species. 

**Figure 4:**
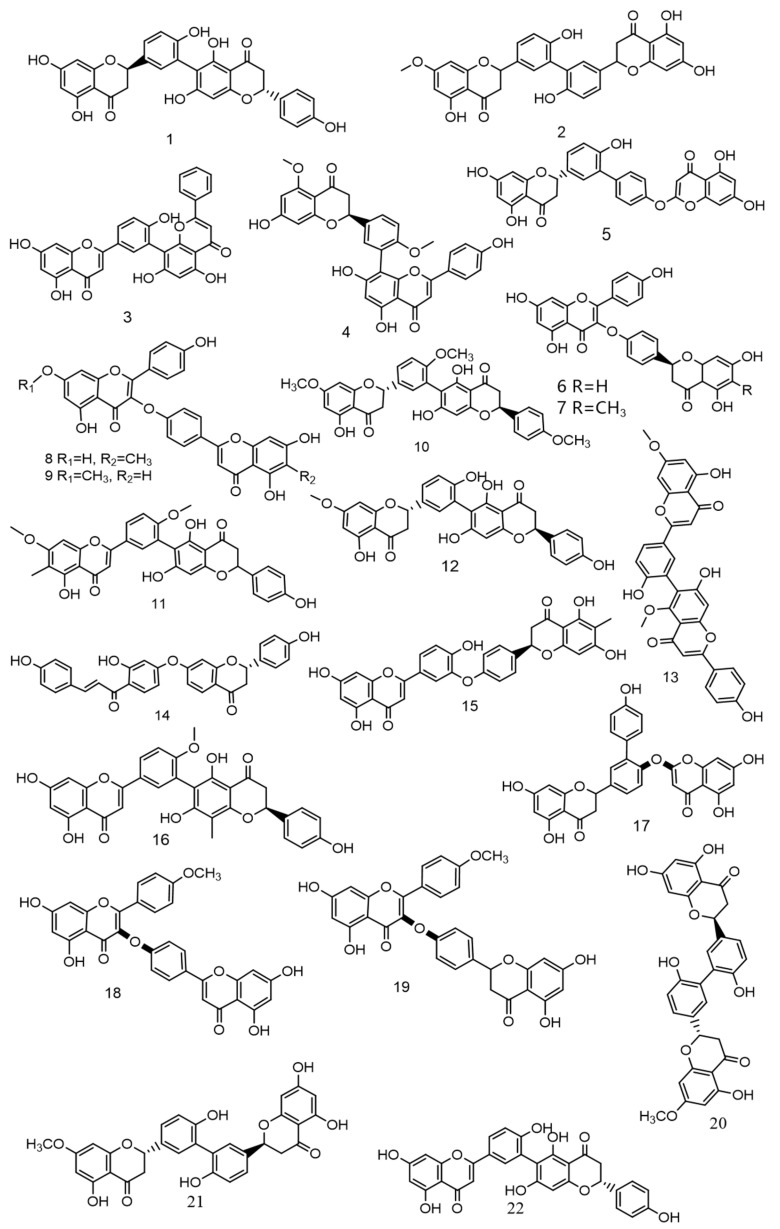
Nine species of bioflavonoids from *Selaginella doederleinii* have been recently discovered.

Over the past 5 years, scientists have discovered numerous novel biflavonoids in *Selaginella* plants [35]. Nine species of bioflavonoids from *Selaginella doederleinii* have been recently discovered [29,30,31]. As shown in Figure 4. Among these, compound **2** [36] showed modest selectivity to A549 cells and MCF-7 cells; compound **4** [37] demonstrated potent cytotoxicity against the human cancer cell lines SMMC-7721, A549, and MCF-7; and compounds **6**–**9** [38] considerably inhibited the proliferation of non-small cell lung cancer. The most active compound, **9**, caused apoptosis and cycle arrest in A549 cells. A new robustaflavone, (2S, 2′S)-2,3,2′,3′-tetrahydrorobustaflavone 7,4′,4‴-trimethyl ether (10), was isolated from a 75% ethanolic aqueous extract of *Selaginella uncinate* [39]. *Selaginella trichoclada* was extracted with 70% ethanol, and five novel biflavonoids were discovered. Among them, compounds **11** and **12** [40] demonstrated moderate cytotoxicity to human cancer cell lines A549 and HepG2; compound **13** [41] was effective against breast cancer via the mitochondrial pathway; compound **14** [42] demonstrated moderate cytotoxicity to human cancer cell lines DU145, MCF-7, and PC3; and compound **15** [43] showed cytotoxicity to the human breast cancer cell line MCF-7. A new biflavonoid (16) was isolated from a 70% ethanolic extract of *Selaginella braunii*, which showed significant antiproliferative effects on SMMC-7721, MCF-7, and A549 cells [44]. From *Selaginella siamensis* and *Selaginella bryopteris*, three novel biflavonoids known as siamamenflavones A–C (**17**–**19**) were isolated. One of them, siamamenflavone B (**18**), exhibited potent inhibition of wild-type EGFR protein [45]. *Selaginella tamariscina* yielded three novel biflavonoids: involvenflavone G (20), H (21), and I (22); H and I may have potent hypoglycemic effects [46].

Biflavonoids derived from plants other than those of the genus *Selaginella* also have significant anti-tumor effects. According to reports, inhibiting Eg5 can cause apoptosis and mitotic arrest. Eg5 inhibitors are therefore becoming the focus of anticancer drug research. As a mitotic kinesin Eg5 inhibitor, morelloflavone from *Garcinia dulcis* binds to the variable site of Eg5, thereby inhibiting the ATPase activity and motor function of Eg5 [47]. Chamaejasmenin isolated from *Stellera chamaejasme* L. roots inhibited the proliferation of cells from eight human solid tumor cell lines (HepG2, SMMC-7721, A549, MG63, U-2 OS, and KHOS, HCT-116 and HeLa) [48]. Japoflavone D, a biflavonoid from *Lonicera japonica* flower buds with significant antioxidant activity, has a dual regulatory effect on the apoptosis of liver cancer cells under various oxidative circumstances [49].

## 3. Synthesis of Biflavonoids

The complex structure of biflavonoids and the similar polarity of different biflavonoids make it very difficult to extract and isolate high-purity biflavonoids from plants. More and more researchers are now synthesizing to obtain biflavonoids with higher purity. Its synthesis is designed and determined according to the way of linkage between two flavonoid molecules. The common synthesis methods are C–C linkage and C–O–C linkage.

Biflavonoids via C–C molecular linkage: Initially, Woo et al. [50] used Ullmann coupling and Suzuki coupling to obtain a natural biflavonoid, ginkgetin, which provided ideas for later proposed more efficient synthesis methods. Park et al. [51] reported the first use of Suzuki reaction to couple flavonoid-3′-boronic acid ester and 4′,5,7-tris amentoflavone by coupling 4′,5,7-trimethoxy-8-iodoflavone. This method can also be used for the synthesis of various other C–C biflavonoids and biphenyl compounds in addition to amentoflavone. In the same year, Nanjan et al. [52] reported a different synthetic method from the conventional Ullmann coupling and Suzuki coupling to obtain biflavonoids by using the principle that ceric ammonium nitrate (CAN) can achieve oxidative dimerization reactions of flavonoid C–C through a single electron transfer mechanism.

Biflavonoids by C–O–C molecular connection: Earlier, Koich et al. [53] synthesized hinokiflavone connected by C4′–O–C8’’ by biphenyl, acylation, and finally condensation utilizing an iodide flavonoid and a flavonoid as the starting materials. The first total synthesis of ochnaflavone was made after Ndoile et al. [54]. The method was based on the reaction of diaryl ethers with 2,4,6-trimethylacetophenone to form chalcone, followed by cyclization and demethylation to obtain the target compounds. As mentioned above, these synthesis methods have many steps and harsh reaction conditions, and we will continue to explore new synthesis methods to improve synthetic yields. For example, more practical catalysts can be used, more efficient equipment can be used to speed up the reaction rate, and the biflavonoids structure can be modified to facilitate the coupling synthesis of biflavonoids.

## 4. *Selaginella* Biflavonoids and Therapeutic Effects on Cancer

### 4.1. Inhibition of Cancer Cell Growth and Proliferation

The cell cycle is the basic process of cell division, which is divided into four successive phases in actively dividing mammalian cells: G1 (gap 1), S (DNA replication), G2 (gap 2), and M (mitotic cellular and nuclear division) phases [55]. Dysregulation of the cell cycle can cause abnormal growth of tumor cells, which will ultimately result in a malignant phenotype of cancer cells [56]. Cyclin-dependent kinases (CDKs), CDK subunits (cell cycle proteins), and CDK inhibitors (CDKI) are three groups of genes that are involved in the cell cycle transition. Together, they comprise a complex regulatory network that coordinates with cellular signaling pathways to underpin cell cycle regulation at the molecular level [57]. SFB has been shown to inhibit cell proliferation by interrupting the cell cycle in numerous in vivo and in vitro experiments.

Lee et al. [58] found that in SiHa and CaSki human cervical cancer cells, amentoflavone downregulated the expression of cell cycle proteins and hyperphosphorylated retinoblastoma (p-pRb) while enhancing the expression of CDKI and p53, thereby arresting the cell cycle in the sub-G1 phase and ultimately inhibiting the growth and proliferation of tumor cells. Not coincidentally, amentoflavone has also been found to be a potential cell cycle inhibitor targeting the cell cycle protein B1 (Cyclin B1), and it induced G1 phase arrest in esophageal squamous carcinoma cells. This discovery was made by Chen et al. [59], who identified potential pivotal nodes as drug targets based on virtual screening of pharmacophores and molecular modeling and evaluated their anti-tumor activity in vitro studies. Recent studies have also found that amentoflavone reduced the expression of cell cycle proteins D1(Cyclin D1), CDK4, and CDK6, thereby arresting the cell cycle in the sub-G1 phase [60]. Additionally, the S-phase may also be a target for SFB. According to Yang et al. [61], hinokiflavone affected the survival of A375 and B16 cells by inducing apoptosis and preventing cell cycle progression in S-phase in a concentration-dependent manner. According to Liu et al. [62], amentoflavone inhibited S-phase kinase protein 2 (Skp2) through the ROS/AMPK/mTOR signaling pathway, contributing to its anti-tumor effect on ovarian cancer. Skp2 was originally identified as a protein that interacted with cell cycle protein A, and it has been reported to regulate a variety of key factors involved in human malignant tumorigenesis and progression, including p21, p57, p130, c-Myc, and E2F1 [63]. Furthermore, according to the study of Lee et al. [64], the G2/M phase may be another significant target for the anti-tumor effects of SFB. Ginkgetin regulates G2 cell cycle arrest induced by genes regulated by B-Myb through the regulation of miR-34a. Cyclin B1, cell division cycle 25C (Cdc25c), and cell division cycle 2 (Cdc2) are among the proteins for which the levels decrease during the G2/M phase. In addition to regulating the expression of genes closely associated with cell cycle arrest, amentoflavone also arrested cells in the G2 phase by perturbing the balance of microtubule dynamics while upregulating the expression of p21 and downregulating the expression of CDK1/2 in SKOV3 ovarian cancer cells [65]. Another study found that hinokiflavone, an inhibitor of MDM2, induced G2/M phase arrest in human colon cancer HCT116 cells through activation of the p53 signaling pathway [66]. The transcription factor is known as the tumor suppressor p53, which is activated in response to stress signals such as DNA damage, oxidative stress, and oncogene activation, and it can restrict cell growth and trigger cell death [67]. Murine double minute 2 (MDM2) functions as the E3 ubiquitin (Ub) ligase targeting p53 for ubiquitination-mediated proteasomal degradation [68]. MDMX is a homolog of MDM2, although lacking E3 ligase activity on its own, and heterodimerizes with MDM2 to increase MDM2-mediated p53 ubiquitination [69,70,71]. For example, recent studies have shown that hinokiflavone inhibits MDM2 activity by targeting the MDM2-MDMX RING structural domain. Once again, the tumor-suppressor gene p53 is inhibited by MDM2 inactivation, which ultimately results in cell cycle arrest [72]. As shown in Table 1.

### 4.2. Inhibition of Tumor Metastasis and Angiogenesis

Tumor metastasis is a multistep process by which cancer cells disseminate long distances from the primary site to form secondary tumors [73] and is regulated by proteins associated with metastasis, such as vascular endothelial growth factor (VEGF), urokinase plasminogen activator (uPA), matrix metalloproteinase 2 (MMP-2), and matrix metalloproteinase 9 (MMP-9) [74]. A variety of mechanisms, including angiogenesis, extracellular matrix (ECM) degradation, and the epithelial-mesenchymal transition (EMT), are implicated in this process. EMT is a cellular process whereby cells lose their epithelial characteristics and gain mesenchymal ones, allowing them to migrate and infiltrate the underlying mesenchyme more effectively. Therefore, this process is strongly linked to tumorigenesis, invasion, metastasis, tumor stemness and resistance to therapy [75]. For example, it has been demonstrated that hinokiflavone significantly inhibited the migration and invasion of breast cancer cells by causing the EMT process to malfunction [76]. Interestingly, Kim et al. [77] discovered that the amentoflavone in *Selaginella tamariscina* is a promising EMT inhibitor of tumor invasion and migration by inhibiting TGF-β and restoring E-cadherin expression in vitro. Additionally, amentoflavone dramatically reduced migration, invasion, and EMT in colorectal cancer by upregulating the expression of miR-16-5p, according to recent research [78]. Six well-known miRNAs of the miR-16 family, miR-15-a/B, miR-16-5p, miR-195, miR-424, and miR-497, have been extensively studied in a variety of cancers, and they are thought to act as tumor suppressors to slow the growth of tumors [79,80,81,82,83]. In addition to the aforementioned Wnt/β-catenin pathway, numerous studies have shown that multiple signaling pathways are also involved in the regulation of EMT, such as the study by Wang et al. [84], which found that sotetsuflavon reversed the inhibition of invasion and metastasis of non-small cell lung cancer A549 cells by EMT through TNF-α/NF-κB and PI3K/AKT signaling pathways.

Importantly, other molecular mechanisms can also inhibit tumor metastasis. uPA stimulates the conversion of plasminogen to plasmin, which breaks down the ECM and activates MMP-2 and MMP-9 when it binds to its receptor (uPAR) [11]. The class of zinc-dependent peptidases known as MMPs can modify the ECM by aiding tumor invasion [85]. For example, Adnan et al. [86], after evaluating the potential antimetastatic properties of the ethanolic extract fraction of *Selaginella tamariscina* in A549 and HCT-116 cells and its possible mechanisms, found that it inhibited the movement and invasion of malignant cells and that this effect might be associated with reduced MMP-2/-9 expression. Moreover, MMP-9 is essential for tumor invasion, metastasis, and angiogenesis. Hinokiflavone has been shown to exert potential anti-tumor effects by inhibiting MMP-9 in human-based pharmacophore modeling and kinetic simulations [87]. Several studies have shown that the anti-cancer properties of numerous SFB species are strongly correlated with their capacity to inhibit MMPs, notably the expression of MMP-2 and MMP-9. As an illustration, almost all C–C biflavonoids have the capacity to regulate MMPs. Hinokiflavone was found to have a similar effect, decreasing the ability of A375 and B16 melanoma cells to invade and migrate, as well as KYSE 150 cells, an esophageal squamous cell carcinoma line, by downregulating MMP-2 and MMP-9 expression [61,88]. Amentoflavone has also been discovered to inhibit invasion and migration of bladder cancer cells by reversing EMT via nuclear factor-kappa B (NF-κB) inactivation and by lowering the expression of MMP-2, MMP-9 and uPA [89]. A family of transcription factors known as NF-κB plays a key role in controlling the development of tumors, the immune system, and inflammation [90]. By overexpressing tumor progression-associated proteins encoded by NF-κB-targeted genes, active NF-κB promoted tumor growth, anti-apoptosis, and angiogenesis [91,92]. In hepatocellular carcinoma (HCC) and glioblastoma, amentoflavone was also found to exert anti-tumor effects by blocking Erk activation and downregulating NF-κB [93,94]. In addition, cathepsin B is a member of the cysteine protease papain superfamily, which is closely associated with the invasion and spread of tumors. Amentoflavone and its derivatives have been found by Pan et al. [95] to have a molecular structure that could attach to human histone B. As a result, Bian et al. [96] hypothesized that amentoflavone is a natural inhibitor that prevents the expression of histone B from lessening the invasion and metastasis of tumor cells in immunodeficient mice.

Angiogenesis is the growth of new capillaries, a process mediated by growth factors, adhesion molecules, endothelial inhibitors, etc. [97]. Tumor growth and metastasis are impacted by dysregulated angiogenesis, which is directly associated with neoplastic disorders [98]. A crucial molecule for the growth and survival of endothelial cells, VEGF can increase vascular permeability, promote endothelial cell proliferation, extravasate plasma fibrin, deposit cellulose, and induce tumor angiogenesis by activating a signaling pathway dependent on the VEGF receptor 2 (VEGFR2) [99]. In vitro, studies have shown that amentoflavone can inhibit angiogenesis and, eventually, apoptosis in MCF-7 cells by suppressing the expression and secretion of VEGF, as well as by inducing the inactivation of NF-κB [100]. Many angiogenesis- and metastasis-related proteins are encoded by NF-ĸB-regulated genes [101]. Moreover, in osteosarcoma U-2 OS cells, amentoflavone exerted the same inhibitory effects on tumor invasion and angiogenesis [102]. As shown in Table 2.

### 4.3. Induction of Cancer Cell Apoptosis

Anticancer drugs can initiate apoptosis, the active pursuit of cell death brought on by specific stimuli, through exogenous and endogenous routes [103]. The intrinsic pathway is closely regulated by the Bcl-2 family of intracellular proteins. This protein family controls the modification of mitochondrial membrane outer membrane permeability by regulating pro- and anti-apoptotic intrinsic pathways [104]. The exogenous pathway involves ligand binding, such as tumor necrosis factor-alpha (TNF-α) and tumor necrosis factor-related apoptosis-inducing ligand (TRAIL), to their corresponding receptors. Upon binding, a death-inducing signaling complex is formed, which activates a series of proteases, including caspase-3, -6, -7, -8, -9, and -10, ultimately resulting in cell death [105]. It has been shown that ginkgetin inhibited human leukemia cell proliferation through the extrinsic pathway by TNF-α signaling [106]. Amentoflavone induces apoptosis in SiHa and CaSki cervical cancer cells by inhibiting the expression of human papillomavirus protein E7, which is achieved through an intrinsic pathway [46]. It has also been demonstrated that ginkgetin activated both exogenous and endogenous pathways in MCF-7 cells, including upregulating the expression of Bax while downregulating the expression of Bcl-2 and survivin and increasing the active production of caspase-8, caspase-9, and caspase-3 in MCF-7 cells [107]. Amentoflavone not only significantly reduced cell viability and activated NF-ĸB but also caused the expression of cellular Fas-related proteins with death domain-like interleukin-1β (LI-1β) convertase inhibitor protein (C-FLIP) and myeloid leukemia 1 (MCL1) proteins. However, it also significantly contributed to the loss of mitochondrial membrane potential and the production of active caspase-3 and -8, which triggered apoptosis through the intrinsic and extrinsic pathways in U-87 MG cells [108]. It was also discovered that amentoflavone caused apoptosis in the human bladder cancer cell line TSGH8301 in a dose-dependent manner through both FAS/FASL-dependent exogenous pathways and mitochondria-dependent endogenous pathways [89].

In apoptosis, reactive oxygen species (ROS) are crucial. Excess ROS can harm DNA, proteins, mitochondria, and endoplasmic reticulum under pathological circumstances, causing cell cycle arrest and apoptosis. Tumor cells are prone to produce more ROS and are, in turn, more vulnerable to ROS than healthy cells due to decreased levels of antioxidant enzymes, which results in cancer cell death [109]. On the basis of this body of theoretical knowledge, delicaflavone was discovered to inhibit PI3K/Akt/mTOR and Ras/MEK/Erk signaling cascades while inducing ROS-mediated cell cycle arrest and death through endoplasmic reticulum (ER) stress and mitochondrial mechanisms [110]. Furthermore, robustaflavone A from *Selaginella trichoclada* promoted MCF-7 cell death by upregulating VDAC2 channels and downregulating Nedd4 E3 ubiquitin ligase, which resulted in ferroptosis and generation of ROS [41]. Hinokiflavone inhibited the growth of colorectal tumor cells, which triggered apoptosis via the ROS-mitochondria-mediated apoptotic pathway. Increased mitochondrial oxidative stress resulted in cytochrome C release, an irreversible process that ultimately resulted in caspase activation and cell death, according to the findings of Zhou et al. [111]. It has also been shown that hinokiflavone promoted apoptosis by increasing mitochondrial reactive oxygen species (mtROS) levels and activating the c-Jun amino-terminal kinase (JNK) pathway [112]. Chen et al. [113] developed hinokiflavone hybrid micelles after confirming the role of hinokiflavone in the mitochondria-dependent intrinsic induction of the apoptotic pathway. The micelles significantly increased the anticancer efficacy of hinokiflavone and were also linked to high levels of ROS-induced reduction of mitochondrial membrane potential and promotion of tumor cell apoptosis. Additionally, ROS functioned as signaling molecules that activated the AMP-activated protein kinase (AMPK) pathway, which inhibited cell proliferation and prevented tumorigenesis. In a similar manner, delicaflavone has been shown to induce apoptosis in HeLa cervical cancer cells through the mitochondrial pathway while blocking the MAPK signaling pathway [114].

Some molecular targets of SFB-induced apoptosis in tumor cells and their role in regulating gene expression have also been identified. Phosphoinositide, a polyphosphorylated derivative of phosphatidylinositol, has been established in numerous studies to be a membrane-bound signaling molecule that is essential for a number of biological processes in cells [115,116,117]. Furthermore, phosphatidylinos itol-3-kinase (PI3K) activates protein kinase B (Akt) by phosphorylating Thr308 and Ser473 (p-Akt) under normal physiological circumstances [118,119]. For example, Guo et al. [88] found that hinokiflavone may lead to apoptosis in esophageal squamous cancer cells by controlling the PI3K/Akt/mTOR signaling pathway. Wang et al. [120] found that amentoflavone induced apoptosis and inhibited glycolysis in glioma cells by targeting miR-124-3p. When compared to normal brain tissues, miR-124-3p expression was markedly down-regulated in glioma tissue. In parallel, fatty acid synthase (FASN) inhibitors are another important target of SFB for inducing apoptosis in tumor cells. According to Lee et al. [121], amentoflavone inhibited the expression of FASN from inducing apoptosis and exerting anti-proliferative effects in human breast cancer cells. They also discovered that FASN, which supports sustained growth and proliferation, was highly expressed in breast cancer, whereas it was expressed at low levels in normal tissues. Further research revealed that amentoflavone could reduce the expression of FASN at both the mRNA and protein levels. Moreover, it can influence Akt, mTOR, and JNK phosphorylation in SKBR3 cells, as well as inhibit HER2 activation [122]. As shown in Table 3.

### 4.4. Induction of Cancer Cell Autophagy

Autophagy is a process of self-degradation in the normal life of a cell. Specifically, it is the process by which certain components of the cell are encapsulated within the cell membrane, forming a vesicle-like structure that is transported to the lysosome for degradation. The discovery of new medications that increase apoptosis by activating autophagy is a novel method for treating cancer, which is being studied further [123]. Earlier, Wang et al. reported that sotetsuflavone caused apoptosis and cycle arrest in A549 cells, as well as prevented their migration and invasion. In subsequent studies, it was discovered that sotetsuflavone induced autophagy in NSCLC cells by blocking the action of the PI3K/Akt/mTOR signaling pathway. This research offered a theoretical foundation for the clinical use of sotetsuflavone and its use as a chemotherapeutic agent in the treatment of NSCLC [84,124,125]. Lou et al. [126] found that ginkgetin induced autophagic death of non-small cell lung cancer cells through p62/SQSTM1-mediated autophagosome formation as well as redox mechanisms. Ginkgetin may have a preference for p62. Through chemical and genetic processes, ginkgetin increased p62 expression, which lowered cell death, lysosomal acidification, and autophagosome formation. These processes were reversed when p62 was knocked down. Hence, p62 may be the prospective target of ginkgetin-induced autophagic cell death. In addition, hinokiflavone induced autophagy through increased LC3-II expression and degradation of p62 [127]. By activation of the AMPK/mTOR-mediated autophagic pathway, ginkgetin may also reduce HG-mediated mesangial cell hyperplasia, oxidative stress, inflammatory response, and ECM accumulation [128]. This showed that the AMPK/mTOR signaling pathway is an effective target for SFB for exerting anti-tumor effects through the autophagic pathway. Another investigation revealed that delicaflavone induced lung cancer cell autophagic death through the Akt/mTOR/p70S6K signaling pathway [129]. Chen et al. [130] showed that amentoflavone-induced autophagy was also associated with the regulation of not only the AMPK/mTOR signaling pathway but the autophagy-related proteins. Park et al. [131] found that amentoflavone lowered the expression of Bcl-2 and stimulated the formation of autophagosomes while increasing the expression of proteins associated with Beclin 1, Atg7, Atg8, and LC3. These studies have demonstrated that induction of autophagy may be one of the key targets for the anticancer effects of SFB. As shown in Table 4.

### 4.5. Reversal of Drug Resistance and Synergistic Effects

Given the synergistic effect of naturally derived compounds and chemotherapy or targeted therapies, their combination tends to improve treatment effects at low doses and minimize potential side effects, thereby improving patient compliance and quality of life. Hence, the use of natural compounds as adjuvant medicines in the treatment of cancer has emerged as a popular research area [132]. Jung et al. [133] investigated the anticancer therapeutic effect of amentoflavone in combination with doxorubicin and found that the aldo-keto reductase family 1B10 (AKR1B10) was chemo-resistant to doxorubicin, while amentoflavone enhanced the anticancer efficacy by inhibiting AKR1B10. Pre-clinical and clinical studies demonstrated that the treatment of oral squamous cell carcinoma using cisplatin in conjunction with other medications was more effective than treatment with cisplatin alone or in a single modality. As compared to postoperative radiation alone, the combination of cisplatin and postoperative radiotherapy significantly increased recurrence-free survival and overall survival in patients with oral squamous cell cancer [134]. For instance, the apoptotic signals caused by cisplatin were enhanced by amentoflavone. Compared to either amentoflavone or cisplatin alone, the combination of the two dramatically boosted the expression of cleaved caspase-3 and promoted cell accumulation in the sub-G1 phase [135]. Cancer cells have the propensity to develop a tolerance to cancer medications, making drug resistance a common issue in cancer chemotherapy. Aldo-keto reductase (AKR), P-glycoprotein (P-gp), multidrug resistance protein (MRP), and other drug export pumps and detoxifying enzymes are examples of representative tolerance proteins involved in chemotherapy resistance [132]. Amentoflavone has previously been shown to sensitize HCC cells to sorafenib by triggering apoptosis [136,137]. A subsequent study by Su et al. [138] found that amentoflavone combined with sorafenib inhibited NF-κB nuclear translocation, NF-κB phosphorylation, and the metastatic ability of U-2 OS cells, leading to apoptosis and inhibition of Erk/NF-κB signaling, sensitizing osteosarcoma cells to sorafenib treatment. Delicaflavone can increase the expression of CHOP and GRP78 proteins through the ER stress pathway, which inhibits cell proliferation and migration, enhanced apoptosis, and cleaved caspase-3 levels, thereby reversing drug resistance in A549/DDP and PC9/DDP cells [139]. It is interesting to note that PARP-1, or polyadenosine diphosphate ribose polymerase-1, has been identified as a potential option for chemosensitizers and anticancer drugs. It has been shown that amentoflavone is a selective PARP-1 inhibitor [140].

### 4.6. Regulation of the Tumor-Associated Microenvironment

At first, cancer was thought to be a genetic or cellular expression disorder, and it was also thought to result from the disturbance of the tumor microenvironment (TME) [141]. ECM, stromal cells, immune cells, and other secreted molecules like growth factors, cytokines, and blood and lymphatic vascular networks coordinate with each other to regulate TME [142,143]. Emerging evidence suggests that SFB regulated the TME primarily by mediating inflammatory responses and immunosuppression [144]. Cheng et al. [145] found that ginkgetin regulated the tumor-associated microenvironment by suppressing the inflammatory response. In HeLa cells, ginkgetin reduced the expression of pro-inflammatory molecules, such as TNF-α, IL-1β, and IL-8 [146]. According to this study, ginkgetin treatment of HeLa cells for 48 h significantly decreased the expression levels of TNF-α, IL-1β, and IL-8 mRNA in a dose-dependent manner. It also significantly decreased the levels of phosphorylation of p38 and NF-κB, which had an anti-proliferative effect on HeLa cells. In addition to being a major exogenous mediator of apoptosis, TNF-α is a cytokine produced mainly by activated macrophages. TNF-α may have pro-apoptotic or anti-apoptotic effects depending on the cellular context [147]. Furthermore, the protective effect of amentoflavone on myocardial ischemia-reperfusion injury in vitro and in vivo through inhibition of apoptosis and inflammation demonstrated that the protective effect of amentoflavone on ischemia-reperfusion injury might be related to the decreased release of proinflammatory factors and the inhibition of the inflammatory response [148]. Amentoflavone also exerts an anti-endotoxin-induced inflammatory effect on BV2 microglia through inhibition of the TLR4/MyD88/NF-κB pathways and activation of the Nrf2/HO-1 pathway [149]. Similarly, Li et al. [150] found that delicaflavone reactivated anti-tumor effects by eliminating monocyte myeloid cell-mediated immunosuppression. Wang et al. [151] demonstrated that delicaflavone inhibited the growth of lung cancer cells by suppressing Mettl3/Mettl14 in order to activate anti-tumor immunity.

## 5. In Vivo Studies

In numerous xenograft models, SFB species have been found to suppress the growth of various malignancies. SFB significantly improved survival and reduced tumor size in target model animals. Through a variety of signaling pathways, the inhibitory effect of SFB on xenograft tumor animal models can be shown through the stimulation of apoptosis, cell autophagy, reduction of cell proliferation, and prevention of metastasis and invasion. Sim et al. [152] used CT-26 tumor xenograft model and a stromal plug assay to study the anti-angiogenic effect in vivo and found that robustaflavone reduced the volumes and weights of CT-26 cell-derived tumors. A significant decrease in blood vessel density was observed in robustaflavone-treated tumors. Robustaflavone was also shown to inhibit VEGF-A-stimulated blood vessel formation in vivo in Matrigel plugs. This suggests that robustaflavone can potentially inhibit tumor growth and metastasis in an angiogenesis-dependent manner. Additionally, Amentoflavon treatment decreased Erk/NF-κB activation and expression of tumor progression-related proteins in U-2 OS osteosarcoma cell xenograft models when compared to solvent-treated groups. This significantly reduced tumor cell invasion and metastasis and induced apoptosis [93,153]. Tsai et al. [136] used in vivo tests to confirm the anti-tumor effects of amentoflavone combined with sorafenib in HCC. They found that the combination significantly increased sorafenib’s ability to inhibit tumor growth and upregulated the expression of anti-apoptotic proteins and Erk/Akt phosphorylation.

The role of SFB in preventing the growth and proliferation of cancer cells through mediating anti-tumor immune responses has also been demonstrated in vivo, in addition to the signaling pathways outlined above. Yao et al. [154] isolated and studied the active anticancer component of SFB using electrospray ionization quadrupole time-of-flight mass spectrometry (HPLC-QTOF-MS). Male C57 BL/6 mice were used in a xenograft model of mouse Lewis lung cancer (LLC), and it was discovered that SFB improved the anticancer immune response. The mechanism by which delicaflavone inhibited the growth of lung cancer cells by activating anti-tumor immunity has also been demonstrated in vivo. The anticancer immunological response mediated by delicaflavone was reversed in tumor-bearing mice by overexpression of the N6-methyladenosine (m6A) transferase Mettl3/Mettl14 [151]. In addition, the pharmacokinetic profile of delicaflavone in rats was also examined by Chen et al. [155] using a validated HPLC-MS/MS method, including its binding characteristics to human serum albumin utilizing multispectral and computer simulation approaches. The results showed that delicaflavone was rapidly eliminated after intravenous administration, with wide tissue distribution. However, it showed linear kinetics in the dose range of 30–60 mg/kg and poor oral bioavailability. The findings highlight the pharmacological tactics that could aid in developing new cancer therapies and in advancing our knowledge of the pharmacokinetics and plasma protein binding characteristics of the prospective anticancer agent delicaflavone. As shown in Table 5.

## 6. Discussion

Despite the fact that a significant amount of scientific research indicates that SFB molecular components, such as amentoflavone and hinokiflavone, have good anti-tumor potential, their poor water and lipid solubility prevents them from being effectively absorbed in the gastrointestinal tract, which restricts their clinical use. Therefore, structural modification and the development of related dosage forms are very effective ways to improve the medicinal value of SFB. For example, Li et al. [156] structurally modified delicaflavone to obtain C4‴-OH (C4‴-acetyl-delicaflavone, 4‴ADLF), resulting in increased water solubility, which was found through a pharmacodynamic in vitro and pharmacokinetic evaluation to have better anticancer activity and bioavailability. To increase their solubility, oral bioavailability, and potency for greater anti-tumor effects, Chen et al. [157] created and refined precursor liposome formulations of the five main anti-cancer active components in SFB extracts. Subsequently, a new oral delivery formulation was developed using solid dispersion technology, again with better solubility, dissolution, and oral bioavailability of the main ingredients, and validated by in vivo studies [158]. It is particularly important to use different techniques to improve the bioavailability of these bioactive biflavonoids to broaden their clinical application prospects. To increase their solubility and bioavailability, Feng et al. [159] prepared amentoflavone-loaded TPGS/Soluplus hybrid nanomicelles for the first time using TPGS and Soluplus as carriers. These carriers are amphiphilic copolymer nanostructures whose hydrophilic tail is exposed and whose hydrophobic head is concealed in the central region. As a result, hydrophobic medicines become more soluble. The in vivo metabolism of amentoflavone has been studied in rats, and the results showed an improvement in the bioavailability of amentoflavone. The use of SFB components as the primary ingredients in combination medications for the treatment of various cancers has also drawn considerable attention due to extensive studies on the anti-tumor activity of SFB and its medicinal value. Recently, Yang et al. [160] invented a combination drug containing amentoflavone, ginkgetin, hinokiflavone, podocarpusflavone A, bilobetin, and 7,4′,7″,4‴-methylsc iadopitysin for the treatment of laryngeal cancer. Mechanistically, the combination of drugs has been shown to induce apoptosis in laryngeal cancer cells by affecting the mitochondrial apoptosis signaling pathway. Downregulation of the phosphorylation levels of STAT3 protein, Akt protein, and NF-kappa B protein resulted in the inhibition of the migratory capacity of laryngeal cancer cells and activation of the mitochondrial apoptotic signaling pathway, thereby inducing apoptosis in laryngeal cancer cells. Of interest is that in the prevention and treatment of breast cancer, combinations of SFB drugs have been reported, and tablets, capsules, or drops containing bilobetin have been prepared with good clinical results [161].

Although SFB has been shown to have remarkable anti-tumor activity, there are no reports related to SFB being marketed as an anti-cancer drug or being tested in preclinical/clinical trials. Additionally, the in vivo bioavailability and safety assessment of the SFB needs to be further studied. However, biflavonoids from other natural plants have been tested in preclinical/clinical trials and have shown promising results. For example, morelloflavone, a biflavonoids from *Garcinia dulcishas*, has been reported for its clinical application in anti-tumor. Lei et al. [162] prepared an ointment based on morelloflavone to be applied topically to breast cancer lumps. Change 2 to 3 times a week. One month is a course of treatment, and surgery is performed after 1 to 2 courses of treatment. The efficacy was determined according to the unified standard, among which 15 cases were significantly effective, 44 cases were effective, and 18 cases were ineffective. The total effective rate was 76.6%, and the significant rate was 19.5%.

The pharmacokinetic study of biflavonoids is still in the exploration stage, mainly for some single components, and different subjects and different drug delivery routes may affect the pharmacokinetic behavior of the active ingredients in vivo. The pharmacokinetic characteristics of amentoflavone in rats were studied by Liao et al. [163] using LC-MS/MS. The results showed that 90.7% ± 8.3% of amentoflavone (300 mg/kg), 73.2% ± 6.29% of amentoflavone (10 mg/kg) and 70.2% ± 5.18% of amentoflavone (10 mg/kg) were detected by these three administrations, respectively. In addition, Shan et al. [164] reported the quantitative analysis and pharmacokinetic study of biflavonoids, including amentoflavone and hinokiflavone, in rat plasma by UFLC-MS/MS for the first time. The results showed that the spiked recoveries of all analytes and IS were greater than 85%. The relative standard deviations for both intra-day and inter-day precision were within 15%, and the RE ranged from −6.6% to 8.0%. These data provide information for further evaluation of their biological efficacy and preclinical development.

To sum up, the conflicting results of SFB in drug development may be due to its poor bioavailability and the lack of efficacy of monotherapies in many diseases. However, problems, such as weak solubility of SFB in water, chemical instability and limited bioavailability, can be remedied by changing the structure and utilizing new delivery systems. Additionally, by enhancing patient compliance and quality of life, innovative dosage forms of therapeutic medications might enhance treatment outcomes, which may have favorable pharmacoeconomic effects for society. Notably, there is sufficient evidence that combining SFB with other bioactive ingredients may produce synergistic therapeutic effects by increasing the bioavailability of SFB and amplifying the metabolic effects of the combined agents. In our subsequent work, we will aim to break through some of the challenges in pharmacokinetics, toxicity, and bioavailability of SFB for clinical use as an anti-cancer drug.

## 7. Conclusions

The anticancer effect of SFB has broad research prospects. Mounting evidence indicates that it exerts its anticancer effects mainly through inhibiting cancer cell proliferation, inducing apoptosis and autophagy, inhibiting tumor cell metastasis and invasion, reversing drug resistance, and regulating the TME. The anti-tumor effects of SFB in vitro and in vivo, as well as its potential modes of action, are all reviewed in detail in this work. Additionally, novel approaches to enhance the biological activity of SFB through water solubility enhancements and medication combinations are presented. To establish its therapeutic efficacy in tumors, as well as its specific effects, it is important to note that the existing clinical trials are still insufficient in breadth and number. Thus, more extensive clinical trials will be required in the future to enable SFB to be widely used in the clinical treatment of cancer for the benefit of patients.

## Figures and Tables

**Table 1:** The effect of SFB on cell cycle arrest and its mechanisms in different cancer cells.

Biflavonoid Type	Cancer Type	Cell Lines	Cell Cycle	Mechanism	References
Amentoflavone	Cervical Cancer	SiHa, CaSki	G(1)	↓ Cyclins and p-pRb	[58]
				↑ CDKI and p53	
Amentoflavone	Esophageal squamous carcinoma	-	G(1)	↓ CyclinB1	[59]
Amentoflavone	Colorectal cancer	HT-29	G(1)	↓ CyclinD1, CDK4andCDK6	[60]
Hinokiflavone	Melanoma	A375, B16	S	–	[61]
Amentoflavone	Ovarian Cancer	SKOV-3, OVCAR-3	S	(-) Skp2 ↓ p21, p57, p130, c-Myc and E2F1	[62]
Ginkgetin	Colorectal Cancer	HCT-116	G2/M	↓ CyclinB1, Cdc25c and Cdc2	[64]
Amentoflavone	Ovarian Cancer	SKOV3	G2	↓ CDK 1/2	[65]
				↑ P21	
Hinokiflavone	Colon cancer	HCT-116	(-)MDM2	(−) P53	[66,72]

In the Table 1, “↓” indicates a decreasing effect; “↑” indicates an increasing effect; “(−)” indicates an inhibiting effect.

**Table 2:** The effect of SFB on cell metastasis and angiogenesis in different cancer cells.

Biflavonoid Type	Cancer Type	Cell Lines	Mechanism	Sign Pathway	Reference
Hinokiflavone	Breast cancer	MDA-MB-231	(−) EMT	–	[76]
			↓ MMP-2/-9		
Amentoflavone	Non-small cell lung cancer	A549	(−) EMT	EMT	[77]
			↓ TGF-β and E-cadherin		
Amentoflavone	Colorectal cancer	HCT-116 and SW480	(−) EMT	Wnt/β-catenin	[78]
			↑ miR-16-5 p		
Sotetsuflavon	Non-small cell lung cancer	A549	(−) EMT	TNF-α/NF-κB and PI3K/Akt	[84]
			↑ E-cadherin		
			↓ N-cadherin, vimentin, and Snail, MMP-9andMMP-13		
SFB	Non-small cell lung cancer, Colorectal cancer	A549, HCT-116	↓ MMP-2/-9	–	[86]
Hinokiflavone	Breast cancer	MCF-7	(−) MMP-9		[87]
Hinokiflavone	Melanoma	A375, CHL-1 and B16	↓ MMP-2/-9	–	[61]
Hinokiflavone	Esophageal squamous carcinoma	KYSE 150	↓ MMP-2/-9	–	[88]
Amentoflavone	Bladder Cancer	TSGH8301	(−) NF-κ B	–	[89]
			↓ MMP-2, MMP-9, and uPA, VEGF		
Amentoflavone	Hepatocellular carcinoma	SK-Hep1	↓ MMP-9, XIAP, VEGF, Cyclin-D1, and pERK	Erk/NF-κB	[93]
Amentoflavone	Glioblastoma	GBM8401	↓ MMP-2, MMP-9, XIAP, cyclinD1 and VEGF	Erk/NF-κB	[94]
Amentoflavone	Colorectal cance	Caco-2/15, CCL-247 and HT29, CCL 229, CCL 228, CCL-227, HEK293T, CCL-21, CCL-222	(−) Cathepsin B	–	[95]
Amentoflavone	Breast cancer	MCF	(−) NF-κ B	–	[100]
			(−) VEGF		
Amentoflavone	Osteosarcoma	U2OS	(−) NF-κ B	–	[102]
			(−) VEGF		

In the Table 2, “↓” indicates a decreasing effect; “↑” indicates an increasing effect; “(−)” indicates an inhibiting effect.

**Table 3:** The effect of SFB on cell apoptosis and its mechanisms in different cancer cells.

Biflavonoid Type	Cancer Type	Cell Lines	Mechanism	Sign Pathway	Reference
Ginkgetin	Chronic lymphoblastic leukemia	K562 cells	↓ TNF-α	TNF-α	[106]
			↑ Caspase-8, Caspase-9 and Caspase-3		
Amentoflavone	Cervical cancer	SiHa and CaSki	↓ Bcl-2	-	[58]
			↑ Bax, Caspase-3/-9		
			(+) PPARγ/PTEN		
Ginkgetin	Breast cancer	MCF-7	↓ Bcl-2, survivin	MAPKs	[107]
			↑ Bax, Caspase-8, Caspase-9, and Caspase-3		
Amentoflavone	Glioblastoma	U-87 MG	↑ LI-1β, C-FLIP, MCL1, caspase-3 and -8	NF-ĸB	[108]
Amentoflavone	Bladder cancer	TSGH8301	↓ MCL-1, FLICE	-	[89]
			↑ FAS, FAS-ligand, Bax		
Delicaflavone	Colorectal cancer	HT29 and HCT116	↑ Caspase-9 Caspase-3, ROS	PI3K/Akt/mTOR and Ras/MEK/Erk	[110]
Robustaflavone A	Breast cancer	MCF-7	↑ ROS	VDAC 2, Nedd 4	[41]
Hinokiflavone	Colorectal Cancer	-	↑ ROS	-	[111]
Hinokiflavone	Hepatocellular carcinoma	SMMC-7721, LO2, HepG2	↓ Bcl-2	mtROS/JNK, NF-κB	[112]
			↑ Caspase-9, Caspase-3, Bax		
Delicaflavone	Cervical cancer	HeLa, SiHa, H8	↓ Bcl-2	MAPK	[114]
			↑ Caspase-9, Caspase-3, Bax		
Hinokiflavone	Esophageal squamous cancer	KYSE150 and TE14	↓ Bcl-2	PI3K/Akt/mTOR	[88]
			↑ Bax, Caspase-3,		
Amentoflavone	Malignant glioma	U87, LV229, U251, LN18 and U373	↓ Bcl-2	ROS/AMPK	[120]
			↑ MiR-124-3p, Bax, Caspase-3,		
Amentoflavone	Breast cancer	SKBR 3	(−) FASN	Akt/mTOR/JNK	[121,122]

In the Table 3, “↓” indicates a decreasing effect; “↑” indicates an increasing effect; “(−)” indicates an inhibiting effect; “(+)” indicates an activating effect.

**Table 4:** The effect of SFB on cell autophagy and its mechanisms in different cancer cells.

Biflavonoid Type	Cancer Type	Cell Lines	Mechanism	Sign Pathway	References
Sotetsuflavone	Non-Small Cell Lung Cancer	A549	-	PI3K/Akt/mTOR	[84,124,125]
Ginkgetin	Non-small cell lung cancer	A549	(−) p62	-	[126]
Hinokiflavone	Chronic myeloid leukemia	K562, LTD	(−) p62	-	[127]
			↑ LC3-II		
Ginkgetin	End-stage kidney disease	Rat glomerular mesangial cells (HBZY-1)	(−) TNF-α, IL-1β, IL-6	AMPK/mTOR	[128]
			↓ Collagen IV, fibronectin, and laminin, EMC		
Delicaflavone	Lung cancer	A549 and PC-9	↑ LC3-II/LC3-I	Akt/mTOR/p70S6K	[129]
	Glioma	U251 and U373	↑ ATG 5, ATG 7, Beclin 1, LC 3BII	AMPK/mTOR/p70 S6 K	[130]
Amentoflavone	Non-small cell lung cancer	A549	↑ Atg7, Beclin1, Atg3 and LC3, p53, p-p21	-	[131]

In the Table 4, “↓” indicates a decreasing effect; “↑” indicates an increasing effect; “(−)” indicates an inhibiting effect.

**Table 5:** The dose and mechanism of SFB to inhibit tumor growth in xenograft models.

Biflavonoid Type	Model Type	Dose	Administration	Mechanism	References
Robustaflavone	CT-26 tumor xenograft model	1 or 5 mg/kg/day		(−) Angiogenesis (−) Metastasis	[152]
				↓ MMP-2, MMP-9, prolyl hydroxylase, lysyl oxidase, VEGF, Erk-1, Erk-2, TNF-α, IL-1β, IL-6,	
Amentoflavone	U-2 OS tumor xenograft model	100 mg/kg/day	i.g	(−) Metastasis,	[93,153]
				(−) Erk/NF-κB signaling	
Amentoflavone	HCC SK-Hep1 tumor xenograft model	10 or 50 mg/kg/day	i.g	(+) Apoptosis, (−) Proliferation	[136]
				(−) Raf/MEK/Erk and PI3K/Akt signaling transduction	
SFB	LLC tumor xenograft model	50 or 150 mg/kg/day	p.o.	(+) Antitumor immune response	[154]
Delicaflavone	3LL in C57BL/6 mice	0.5mg/kg/day	i.h.	(+) Antitumor immune response	[151]
				(−) Mettl 3/Mettl 14	

In the Table 5, “↓” indicates a decreasing effect; “↑” indicates an increasing effect; “(−)” indicates an inhibiting effect.; “(+)” indicates an activating effect.

## Data Availability

Data sharing is not applicable.
